# Resistance and Biodegradation of Triclosan and Propylparaben by Isolated Bacteria from Greywater

**DOI:** 10.3390/jox15020056

**Published:** 2025-04-15

**Authors:** Daniella Itzhari, Joseph Nzeh, Zeev Ronen

**Affiliations:** Zuckerberg Institute for Water Research, The Jacob Blaustein Institutes for Desert Research, Ben Gurion University of the Negev, Beersheba 8499000, Israel; vanderro@post.bgu.ac.il (D.I.); nzeh@post.bgu.ac.il (J.N.)

**Keywords:** triclosan, propylparaben, antibiotic resistance, phenotypic analysis

## Abstract

We investigated the relationship between antibiotic-resistance genes and the antimicrobial agents, triclosan (TCS) and propylparaben (PPB). The greywater microbiome was repeatedly exposed to triclosan and propylparaben and the effect was analyzed using a combination of PCR, Etest, Biolog, 16S rRNA sequencing, and liquid chromatography. The taxonomic identification points to very similar or even identical isolates, however, the phenotypic analysis suggests that their metabolic potential is different, likely due to genomic variation or differences in the expression of the substrate utilization pathways. For both triclosan and propylparaben, the antibiotic resistance levels among isolates remain consistent regardless of the exposure duration. This suggests that antibiotic-resistance genes are acquired rapidly and that their presence is not directly proportional to the level of micropollutant exposure. In a biodegradation test, TCS was reduced by 50% after 7 h, while PPB decreased only after 75 h. For TCS, the minimal inhibition concentration (MIC) ranged from 64 to above 256 mg/mL. Conversely, for PPB the MIC for the tested strains ranged between 512 and 800 mg/mL. This study highlights the complex interaction between household xenobiotics, greywater microorganisms, and the emergence of antibiotic resistance.

## 1. Introduction

It is already established that greywater is contaminated with pathogens, emerging manmade micropollutants (EMPs), and antimicrobials [1]. Recent research identified the occurrence of both bacteria and EMPs in wastewater that are responsible for the accumulation of antibiotic-resistant bacteria (ARB) [2,3]. Unlike antibiotics, the mode of action of EMPs can be unspecific and target different processes or sites in bacterial cells [4,5]. The same adaptive mechanisms that can create bacterial resistance to antibiotics can act against EMPs. On a global scale, EMPs are used at the household level in much larger quantities than antibiotics. Some of the most detected EMPs in domestic greywater are triclosan (biocide), methylparaben and propylparaben (preservatives), galaxolide and tonalide (fragrances), as well as oxybenzone and octocrylene (UV filters) [6], and benzalkonium chloride [7] (see Supplementary Materials, Table S1 for chemical specification). Although EMPs are toxic to microbes, the biodegradation process is still the dominant process for their removal from the environment in general and greywater in particular [8,9]. Recently, the risk assessment of compounds released from greywater categorized triclosan as a high-priority substance and propylparaben as a medium-priority substance based on their risk quotient [10]. Therefore, this study aimed to describe the characteristics of microbial isolates from greywater exposed to two specific EMPs (triclosan and propylparaben), focusing on the potential changes in susceptibility to antibiotics.

### 1.1. Resistance to Triclosan and Propylparaben

Triclosan (TCS), 2-(2,4-dichlorophenoxy)-5-chlorophenol, is a common antimicrobial agent in many personal care products (soaps, sanitizers, and toothpaste) [11] and is widely detected in aquatic environments at the μg/L to mg/L level [12,13,14]. Its occurrence in the environment is of concern because multiple reports have demonstrated the role of TCS in causing a multiple mechanism response in bacteria. TCS has endocrine-disrupting capabilities [15] and can serve as an external pressure to co-select for antibiotic resistance in many types of bacteria [16,17,18]. TCS induces oxidative stress, causing genetic mutations in a few bacterial genes, such as *frdD*, *soxR*, *marR*, *acrR*, and *fabI* [19,20]. The genes *frdD*, *soxR*, *marR*, and *acrR* are linked to membrane permeability and efflux pumps [21]. The gene *fabI* is an acyl carrier protein reductase gene, which encodes a key enzyme in fatty acid synthesis in bacteria [22]. Some Gram-positive bacteria exhibit an intrinsic resistance to TCS by using exogenous lipids to avoid inhibiting fatty acid synthesis [23]. Sub-inhibitory concentrations of TCS reduce *Escherichia coli* susceptibility to ciprofloxacin, kanamycin, and gentamicin to varying degrees [24]. One study reported that TCS-resistant strains could tolerate up to 500-fold higher antibiotic concentrations than non-TCS-resistant strains [25]. Triclosan increased pathogens’ tolerance to bactericidal antibiotics by up to 10,000-fold in vitro and reduced antibiotic effectiveness by up to 100-fold in a mouse [26]. *Stenotrophomonas maltophilia* exposed to TCS reported reduced susceptibility to chloramphenicol, tetracycline, and ciprofloxacin, caused by overexpression of the multidrug efflux pump SmeDEF [27]. *Salmonella enterica* exposed to increasing TCS concentrations showed a decreased susceptibility to chloramphenicol, tetracycline, and ampicillin, with overexpression of the efflux pump, suggesting the further co-selective potential of antibiotic microbial resistance [28]. Additionally, the transcription of genes encoding β-lactamases and multidrug efflux pumps is significantly upregulated, while the expression of membrane permeability-related genes is reduced [29].

Parabens, para-hydroxybenzoates, or esters of 4-hydroxybenzoic acid are widely used as antimicrobial agents in many foods, pharmaceutical, and cosmetic products due to their excellent antimicrobial activities and low toxicity [30]. Parabens are more active toward Gram-positive than Gram-negative bacteria [31,32]. Studies have linked paraben resistance to cell wall characteristics and nonspecific efflux systems [33]. The effectiveness of parabens correlates with their chemical size, with propylparaben (PPB) being more active against most bacteria than methylparaben [34]. Parabens induce the permeabilization of bacterial membranes, causing the release of potassium [35]. Resistance to parabens has been reported, for example, in the specific strains of *Pseudomonas aeruginosa*, *Burkholderia cepacia*, and *Cladosporium resinae* [30].

### 1.2. Mechanisms of Co-Selection

Co-selection occurs when resistance to one antibacterial agent is accompanied by resistance to another. This occurs when genes encoding resistant phenotypes are co-located on the same genetic element, such as a plasmid, transposon, or integron [36]. There are different types of co-selection: (i) Cross-resistance, whereby one biochemical system can confer resistance to both antibiotics and EMPs; (ii) Co-resistance, whereby resistance to one pollutant results in resistance to other pollutants; (iii) Co-regulatory resistance, whereby various regulatory systems are transcriptionally linked, thus, exposure to one pollutant can lead to resistance to another pollutant by an unknown or unrecognized pathway [37]. Resistance can be either natural, acquired, or adapted. Natural resistance can either be intrinsic (always present in the species) or induced (where genes naturally found in the bacteria are only expressed to resistance levels after antibacterial exposure) [38]. Acquired resistance refers to the resistance that occurs when a bacterium, which is previously sensitive to an antibacterial, develops resistance through either a mutation or the gain of new genetic material from an external source via horizontal gene transfer (HGT) [37]. Adaptive drug resistance is defined as the capacity of bacteria to respond quickly to environmental changes, but once the inducing signal is no longer present, the bacteria typically revert to their original susceptibility to the antibacterial [39]. Table 1 presents identified mechanisms of bacteria potentially conferring co-selection to antibacterial resistance.

The objective of this study is to investigate if exposure to TCS and PPB might induce antibiotic resistance. This study is the first to report on the complex relationship between bacterial resistance to antibiotics caused by antimicrobial chemicals and the biodegradation of antimicrobial chemicals by bacteria. We suggest that exposure to TCS and PPB in greywater, even at a low concentration, will lead to the selection of resistant bacterial strains. We tested the following hypotheses: (1) TCS- and PPB-induced antibiotic resistance is inherited and correlates with the concentration of antimicrobial chemicals; and (2) isolates with antibiotic resistance acquired through co-selection can rapidly degrade TCS and PPB.

## 2. Materials and Methods

### 2.1. Exposure Experiment

We aimed to isolate bacterial strains from household wastewater that can use triclosan and propylparaben as an energy source. Raw greywater was sampled from several different households located in Midreshet Ben Gurion, Israel [41] in October 2022. The greywater was mixed and transported to the laboratory within four hours [42]. A total of 100 mL of sample were centrifuged, to concentrate on the microbial community, and the pellet was resuspended in 10 mL of synthetic greywater. Synthetic greywater was composed of the ingredients listed in Table S2 (Supplementary Material). In total, 1 mL of this cell suspension community was inoculated into a 50 mL falcon tube containing 9 mL synthetic GW, exposed to concentrations of either TCS or PPB, ranging from a sub-MIC to a near lethal concentration (0.02 and 0.2 mg/L, which are environmentally relevant concentrations, and 2 mg/L). It was incubated in triplicates at 25 °C and shaken at 150 rpm. Every 72 h, 1 mL of the cell mixture was transferred to a new falcon tube containing 9 mL synthetic GW with the same respective concentrations of a micropollutant (MP). The transfers were repeated for ten subculture cycles. At the end of the 5th and the 10th transfer cycles, 100 μL were diluted serially in 9 mL of sterilized 0.01 M phosphate-buffered saline (PBS) (pH 7.4). The estimation of the number of viable, culturable heterotrophic bacteria was performed using a heterotrophic plate count (HPC). In total, 0.1 mL from the dilutions were spread on sterile BD Difco™ Plate Count Agar plates and incubated at 30 °C for 18 h. We maintained an MIC concentration for both TCS and PPB 1.2 mg/L for our research. Dilutions were also plated on Mueller–Hinton agar (Sigma-Aldrich, Rehovot, Israel) with different concentrations of micropollutants (control, 1 MIC, and 2 MIC; related to each different compound) at 30 °C for 18 h to screen for resistant bacteria, and the colony forming units were counted. The number of colonies with a treatment was divided by the total bacterial count to calculate the mutation frequency. From the 2 MIC MH plates, bacterial strains were isolated, genomic DNA was extracted from the isolated strains (Gene Elute, Sigma-Aldrich, Rehovot, Israel), and these were identified based on their 16S rRNA gene sequence [43]. All the sequences were deposited in GenBank under accession numbers PQ73249 to PQ73267. The isolates were preserved as 15% glycerol stocks at −80 °C.

### 2.2. Antimicrobial Susceptibility Testing

Mueller–Hinton agar plates were inoculated uniformly on the entire surface with the bacterial isolate (OD of 0.1 at a wavelength of 600 nm) using a sterile cotton swab. The Etest^®^ (BioMérieuxl, Marcy-l’Étoile, France) strips were placed on the plates and incubated at 37 °C for 24 h. After the incubation period, we observed the plates for the growth of the bacterial isolates and the presence of elliptical inhibition zones around the Etest^®^ strips. We determined the minimum inhibitory concentration (MIC) of the elliptical inhibition zone using the Etest^®^ strips. Six different antibiotics were tested, including tetracycline, gentamicin, trimethoprim/sulfamethoxazole, ciprofloxacin, ampicillin, and amoxicillin. Their mode of action that ultimately leads to bacterial cell death is shown in Table 2. The results were interpreted as susceptible, intermediate, or resistant. For the quality control, *E. coli* (ATCC 25922) and *Pseudomonas putida* (ATCC 12633) were used.

### 2.3. Triclosan and Propylparaben MIC Testing

All the isolates were first tested for the MIC of TCS using the agar dilution assay. Bacterial cultures were regrown overnight in a Mueller–Hinton (MH) broth and then diluted in an MH medium to an optical density (OD) of approximately 0.1. A 5 µL aliquot of the bacterial suspensions was spotted on the surface of MH agar plates containing serial dilutions of TCS ranging from 2 to 32 µg/mL (prepared from a 10 mg/mL stock in DMSO). The agar plates were allowed to dry for 15 min and then incubated at 30 °C for 16 to 20 h, after which bacterial growth was observed. The growth kinetics of selected strains at different concentrations of TCS were determined using the broth dilution method. The cultures were pre-grown in MH overnight and then diluted in MH containing TCS at concentrations ranging from 0 to 256 µg/mL, aiming for an OD of approximately 0.1. In total, 1 mL of each concentration was dispensed (triplicates) into a 48-well microplate. The plate was shaken at 50 rpm, and the OD readings were taken with a Tecan Infinite^®^ 200 PRO plate reader (Tecan, Männedorf Switzerland).

### 2.4. Detection of ARGs

The total genomic DNA of the isolates was analyzed using PCR to identify the bacterial and antibiotic resistance genes. PCR was performed with 20 µL reaction volumes composed of 10 µL Ready-Mix for PCR (Biolab, cat. 9597 58026540) and qPCRBIO SyGreen blue mix lo-rox (PCR biosystems, London, UK), 1.6 µL of the respective primers (forward and reverse), 7.4 µL of nuclease-free water, and 1 µL of template DNA. The PCR cycling conditions for all the reactions were as follows: 30 cycles composed of a 5 min denaturation at 95 °C, 1 min annealing at 60 °C, and 30s polymerization at 72 °C. The amplicons were analyzed using gel electrophoresis (in 2% agarose in TAE buffer), stained with ethidium bromide, and visualized under a UV light. The following genes were selected for the PCR analysis: *blaTEM*, *qnrS*, *sul1*, *intI1*, *tetG*, *FabVas*, and *FabVas2*, which are genes coding resistance to antibiotics of the group beta-lactam, quinolone, sulfonamide, tetracycline, and antibiotics that target fatty acid biosynthesis [41]. The results of the PCR analysis will indicate the antibiotic-resistant genes that are present in the obtained isolates; Table S4 (Supplementary Materials) gives an insight into their mechanism of action [50].

### 2.5. Metabolic Activity of the Microbial Isolates

The Biolog Microplate was found to be suitable and convenient for the substrate utilization studies of the closely related microbes [51]. Therefore, we applied the Biolog^®^ GEN III Microplates for both Gram-negative and Gram-positive bacteria (Biolog Hayward, CA, USA). These microplates contained 95 different substrates in wells and controls. The substrates were classified by the type of chemical compound, for example, carbohydrates, amino acids, carboxylic acids, amines, amides, and polymers. Additionally, each well contained a colorless tetrazolium dye. The isolates were shaken in a GEN III Inoculating Fluid solution and then dispersed into the plate wells according to the manufacturer’s instructions. The plates were incubated at 37 °C, and during incubation, the isolates oxidized the substrates in the plate wells and simultaneously reduced the colorless tetrazolium dye to a violet formazan. The color development was measured at 600 nm every 8 h using a Tecan Infinite^®^ 200 PRO plate reader. The rate of utilization of different substrates by different isolates varies, so one can observe a high variability in the rate of color development and its intensity depending on the isolate’s biochemical characteristics, providing a “metabolic fingerprint”. Increased respiration causes a reduction of the tetrazolium redox dye, forming a purple color and thus a higher OD value. The negative control well (no-carbon substrate) was used for the analysis by dividing each well OD by the negative control. The positive control well (A-10) was used as a reference for the chemical sensitivity assays.

### 2.6. Biodegradation Experiment

Based on the results of the 16S rRNA sequencing, isolate D7 from PPB exposure was identified as the most similar to *Pseudomonas* sp. BT5A (Table 3), and isolate D1 from TCS exposure, identified as the most similar to *Achromobacter insolitus* (Table 3), were used for the degradation experiments. Microcosms were constructed using sterile Erlenmeyer flasks with 50 mL of Mueller–Hinton media. The microcosms were inoculated with the isolates (initial 0.1 OD) and were spiked with either TCS or PPB at the concentration of 2 mg/L (controls were run in parallel). Samples were taken every 7 h until the compound was undetectable by the HPLC (UHPLC focused, Dionex UltiMate 3000; Thermo Fisher Scientific, Waltham, MA USA). The samples were filtered (0.22 μm) before the analysis. The HPLC analysis used a Phenomenex, Luna C-18 column analytical column with the dimensions 250 mm × 4.6 mm, 5 μm (Phenomenex Torrance CA, USA). The column temperature was set at 40 °C, and the flow rate was set at 1.0 mL min^−1^ with methanol: DDW (70:30 *v*/*v*) as the mobile phase. The detection wavelength of TCS was 280 nm, and the HPLC mobile phase analyzed 10 μL of the sample extract.

### 2.7. Strains Similarity Analysis

Based on the 16S rRNA sequences and the carbon utilization (Biolog^®^ plates, Hayward, CA, USA, a similarity analysis of the 16 strains was determined [52]. The maximum likelihood method and the Tamura–Nei model were applied for the 16S rRNA gene sequence analysis. For the cluster analysis based on data using Biolog^®^ GENIII plates, we used the metabolic response measured after 24 h. The data were corrected against the negative control, and the clustering analysis (Bray–Curtis similarity) was performed using PAST version 4.17 [53].

### 2.8. Statistical Analysis

The significance (*p* < 0.05) was assessed using the *t*-test in the R software. To visualize data, we used the ggplot2 package in the R software [54]. All the statistical analyses were performed in the R software v3.6.2 unless otherwise stated.

## 3. Results

**Figure 1:**
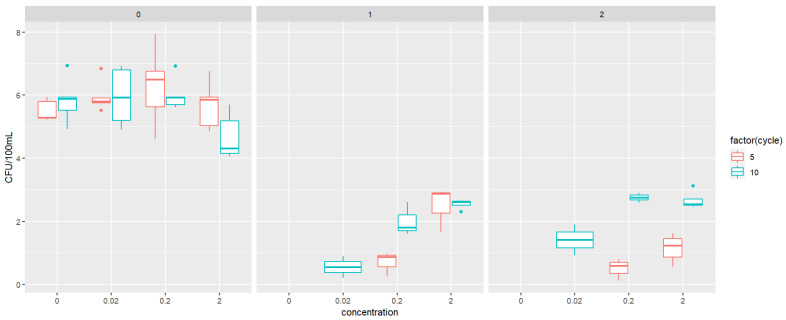
Abundance of colony forming units (CFUs) per 100 mL (in log_10_), plated on either 0MIC, 1MIC, or 2MIC plates, for four different treatments of triclosan exposure (0, 0.02, 0.2, and 2 mg/L).

**Figure 2:**
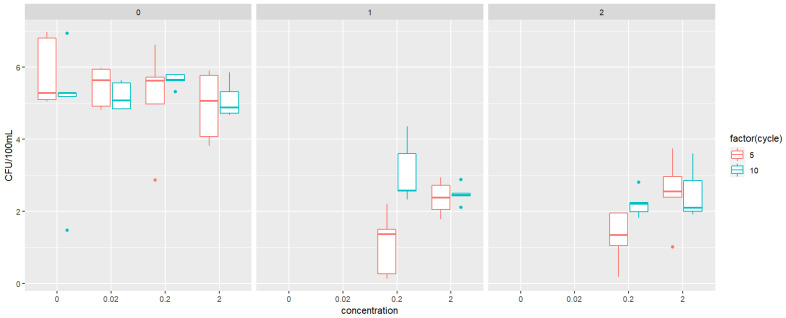
Abundance of colony forming units (CFUs) per 100 mL (in log_10_), plated on either 0MIC, 1MIC, or 2MIC plates, for four different treatments of propylparaben exposure (0, 0.02, 0.2, and 2 mg/L).

Exposure to triclosan and propylparaben at 0.2 and 2 mg/L induced inhibitory effects on bacterial growth, reflected by the total cell concentration that was reduced from 1.22 × 10^7^ CFU/100 mL (0 mg/L dose) to 4.54 × 10^6^ CFU/100 mL (0.2 mg/L dose) and 6.36 × 10^5^ cells/100 mL (2 mg/L dose treatment), respectively. There was no bacterial growth on the 1MIC and 2MIC plates with the treatment of 0 mg/L (control treatment) for the bacterial culture exposed to TCS (Figure 1) and PPB (Figure 2). We observed a significant difference (*p* < 0.05) in the abundance between the 5th cycle and the 10th cycle on the plates for TCS (*p* = 0.0159) and PPB (*p* = 0.0337).

Three strains grown on triclosan were identified as most similar to *Achromobacter insolitus*, *Pseudomonas nitroreducens*, and *Pseudomonas* sp. AU2510. The different propylparaben strains that were isolated had a high similarity, based on the 16S rRNA gene, to *Pseudomonas* sp. BT5A and *Pseudomonas nitroreducens.* Research on isolating from residential soils also reported bacterial strains of the genus *Achromobacter* and *Pseudomonas* [55]. The genus *Pseudomonas* is a Gram-negative, rod-shaped group of bacteria commonly found in the environment [56]. *Pseudomonas* strains have the remarkable capacity to degrade a wide range of substrates [57] and exhibit an intrinsic resistance to many different antimicrobial agents through expressing several efflux pumps that confer multiple drug resistance [58]. *P. nitroreducens* normally inhabits soils, especially those contaminated with oil brine. It is best known for use in the industrial production of polyesters from medium-chain-length fatty acids. *P. nitroreducens* is not considered a human pathogen [59]. However, *P. nitroreducens* (accession number MT472129.1) was previously isolated from greywater on ESBL media, confirming its potential for resisting beta-lactam antibiotics [43]. The strains *Pseudomonas* sp. *BT5A* and *Pseudomonas* sp. *AU2510* have not been described in previous scientific work to the best of our knowledge.

### 3.1. Comparing Actual Resistance (Etest) with ARG Existence

The results of the Etest were interpreted according to the MIC breakpoints published by the CLSI [60,61]. For most of the obtained isolates, no inhibition zone was observed for trimethoprim/sulfamethoxazole. In the isolates obtained from the TCS plates, the genes *qnrS* and *sul1* were present in all the samples, while *blaCTX* was not observed. Most isolates were resistant to gentamicin. There was no significant difference between the isolates obtained in cycle 5 and cycle 10. Most of the isolates obtained from the PPB plates are resistant to ampicillin. Only isolate 11 did not show resistance to any antibiotic, which is remarkable because this is the only isolate with all the antibiotic-resistant genes present. For both triclosan and propylparaben, there is no significant difference in antibiotic resistance patterns between the isolates obtained in cycle 5 and cycle 10. This suggests that antibiotic resistance is obtained when exposed to the micropollutant and long-term exposure does not increase resistance to more antibiotics. In addition, the abundance of ARGs in the isolates is not proportional to the amount of the micropollutant that the isolates were exposed to. There is no noticeable correlation between the number of ARGs and the actual resistance to antibiotics as tested using Etest. Also, no distinguished connection between the specific ARGs and the Etest results could be detected. This observation can be explained by the fact that multiple resistance mechanisms can exist for one antibiotic. For example, molecular mechanisms of tetracycline resistance include efflux pumps, ribosome protection proteins, reduced permeability, ribosomal mutations, and enzymatic inactivation [62]. The *tetG* gene tested in our study is one of the ARGs that is involved in the reduced permeability of the compound and, therefore, does not represent the actual resistance mechanisms of the Etest. The results of the quality control are shown in Table S3 (Supplementary Materials).

### 3.2. Similarity Analysis of the Isolates

**Figure 3:**
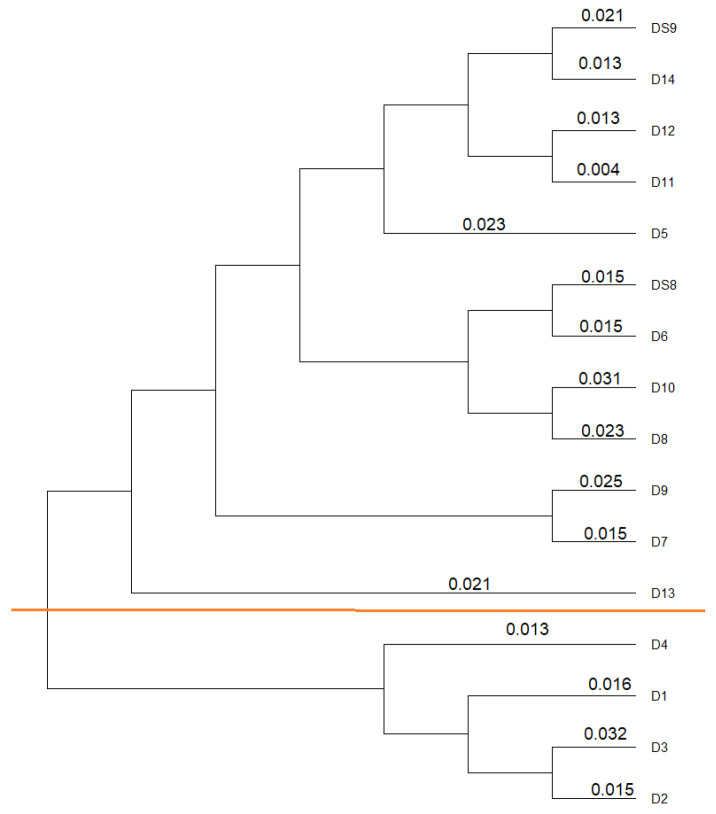
Similarity analysis of the isolates based on the 16S rRNA gene. Below the orange line are the isolates of *Achromobacter*, and above the line are isolates of *Pseudomonas*. The number of each line indicates the similarity.

According to the 16 rRNA gene sequences, the isolates are very similar to each other, though a clear distinction can still be observed between *P. nitroreducens* and *A. insolitus*, as displayed in Figure 3.

### 3.3. Characterization of Isolates Using a Carbon Source Utilization Pattern

**Figure 4:**
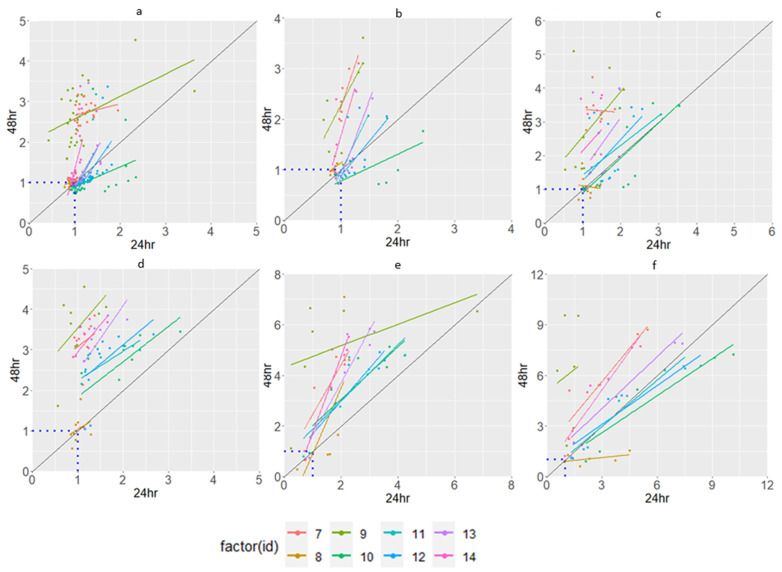
Catabolic activity of isolates exposed to propylparaben, showing: (**a**) sugars; (**b**) carbon utilization; (**c**) hexose acids; (**d**) amino acids; (**e**) carboxylic acids, testers, and fatty acids; (**f**) GN-GP. The figures show the OD value of a specific well divided by the OD value of the negative control. The dotted line at value 1 indicates the similarity between the negative control and the substrate. The diagonal line shows the relationship between the substrate utilization after 24 and 48 h. Dots below the diagonal line are substrates utilized less after the first 24 h.

**Figure 5:**
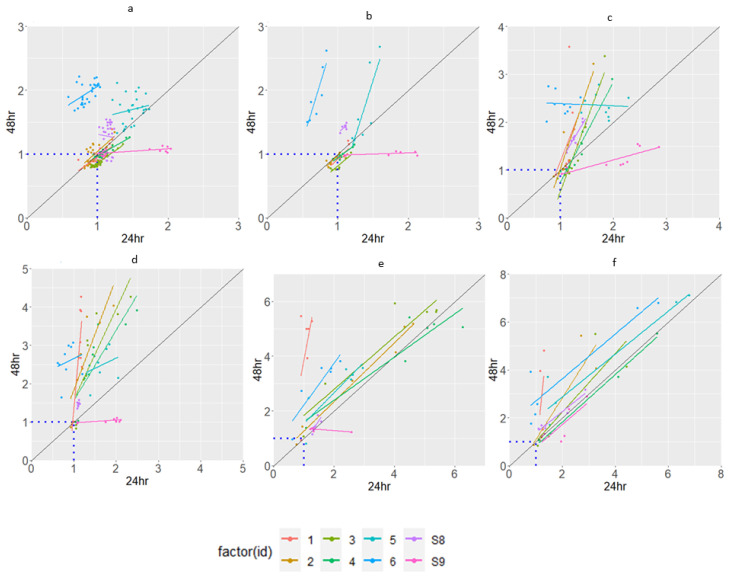
Catabolic activity of isolates exposed to triclosan, showing: (**a**) sugars; (**b**) carbon utilization; (**c**) hexose acids; (**d**) amino acids; (**e**) carboxylic acids, testers, and fatty acids; (**f**) GN-GP. The graph is explained in Figure 4.

The 16S rRNA data suggest that some of the isolates are identical. However, both the ARG patterns and the Etest indicated differences in the phenotypes. Thus, because the rate and extent of the utilization of different substrates by different phenotypes vary, one can observe variability in substrate utilization as a “metabolic fingerprint” determined using the Biolog^®^ assay. To compare the strategies of substrate consumption by microbes, the carbon sources were classified into six main groups: sugars, carbon utilization, GN-GP, carboxylic acids, amino acids, and amines. The isolates showed significant differences in their substrate utilization profiles, as depicted in Figure 4 and Figure 5.

Both isolates exposed to PPB and isolates exposed to TCS minimally utilize the carbon sources classified as “sugars” and “carbon utilization”. Isolate 10 and isolate 9 show a decreased utilization of most substrates when incubated for more than 24 h. Isolate 9 (PPB-exposed) and isolate 1 (TCS-exposed) have the highest utilization of most of the carbon sources. The main variation between the isolates is shown in the utilization of the carbon source “GN-GP, Hexose-PO4, reducing power”. PPB-exposed isolates utilize carbon sources like fatty acids almost in the same quantity. TCS-exposed isolates, however, are sometimes very distinct in their utilization.

**Figure 6:**
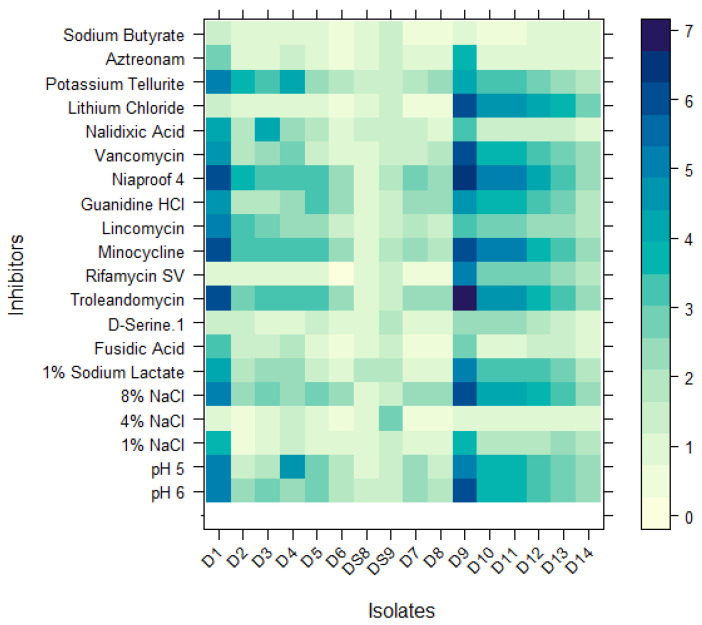
Response to the chemical inhibitors of the Biolog^®^ plate. Yellow colors (near 0) represent no response (inhibition).

When only studying the carbon utilization after 24 h in the heatmap analysis (Figure 6), similar carbon utilization can be noticed with isolates 2, 3, and 4, and with isolates 10, 11, 12, and 13. Isolate 6 has the lowest carbon utilization for most wells compared with the control. No distinction can be found between the isolates obtained after 5 cycles and 10 cycles.

When looking at the resistance to inhibitory chemicals, isolate 12 and isolate 13 react similarly to the same inhibitors, though all the other isolates have a different pattern. The wells with tetrazolium violet and tetrazolium blue had the most activity; both substances are from the class “reducing power”. Tetrazolium is mainly used to indicate extracellular redox activity and cell redox potential [63]. Fast-growing bacteria adapted to high substrate concentrations mostly contribute to tetrazolium dye reduction on bacterial plates [64]. Isolate S8 has resistance to most of the inhibitors.

**Figure 7:**
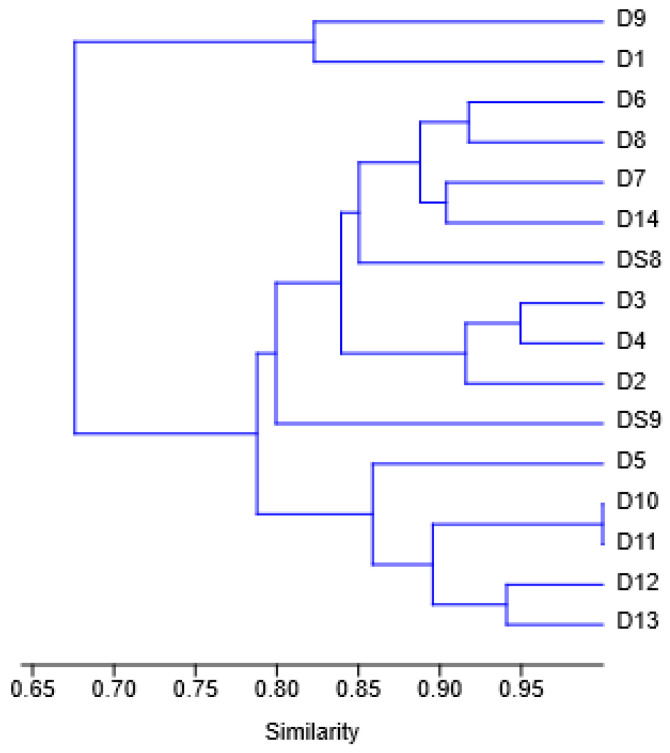
Dendogram based on DNA sequencing, showing the similarity between isolates.

When looking at the similarities of the isolates with a dendrogram, see Figure 7 below, no distinction can be made between the species, unlike the dendrogram based on DNA sequencing.

Overall, while the taxonomic identification points to very similar or even identical isolates, the phenotypic analysis suggests that their metabolic potential is different, likely due to genomic variation or differences in the expression of the substrate utilization pathways.

### 3.4. Resistance and Biodegradation of Triclosan and Propylparaben

We selected strains D4, D7, and D13 to determine the 50% inhibition concentrations (IC_50_) and MIC of both compounds from 24 h growth curves (Supplementary Figures: Figures S1–S6). The TCS MIC was 64, 128, and >256 mg/mL for strains D4, D7, and D13, respectively. Likewise, the PPB MIC was 512, 512, and 800 for strains D4, D7, and D13, respectively. Fitting the IC_50_ values using the OD 24 h as the response value did not always agree with the actual MIC because of outlier OD values at lower concentrations. The high MIC values of strain D13 coincide with the earlier observation of the investigation of antiseptic efficacy under standardized and harmonized conditions, where the MIC for triclosan for *Pseudomonas aeruginosa* (ATCC 15442) was above 512 mg/L [65]. Interestingly, despite the high resistance to TCS by the tested strain, multi-drug resistance was limited (Table 4) because TCS induces resistance to other membrane-active agents but not to clinically relevant antibiotics that target other cell components [66]. 

**Figure 8:**
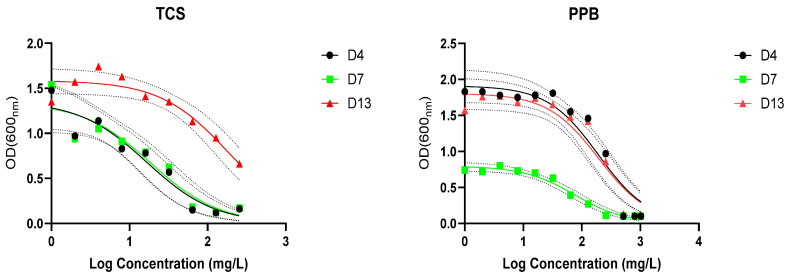
Growth (OD600) of the selected isolates after 24 h in response to an increasing concentration of either triclosan or propylparaben. Dots represent the actual data, the solid line represents the nonlinear fitting of log(inhibitor) vs. response (three parameters model), and the dashed lines are 95% confidence intervals to the fitted lines.

Parabens, including propylparaben, have been found to be effective in low concentrations against fungi and bacteria. Like TCS, they induce cytoplasmic membrane damage. The reported MIC for bacteria ranged between 0.125% for *Bacillus cereus* to 0.8% for *Pseudomonas aeruginosa* [67]. These values are higher than those found in our study, ranging between 0.0512 and 0.08%. No clear co-resistance trend for antibiotics was observed in our strains, where D4 is sensitive, D7 is resistant to gentamicin, and strain D13 is resistant to amoxicillin and ampicillin (Table 3). The data indicate that the antibiotic resistance in these strains is not related to paraben exposure. Earlier studies suggested that parabens in wastewater effluents can induce ARG abundance in the river biofilm community [68]. While in greywater we found multiple ARGs in influent, filter bed, and effluent; we could not relate it to the presence of either triclosan or parabens in the greywater [41,42,43] (Figure 8).

### 3.5. Biodegradation of Triclosan and Propylparaben

**Figure 9:**
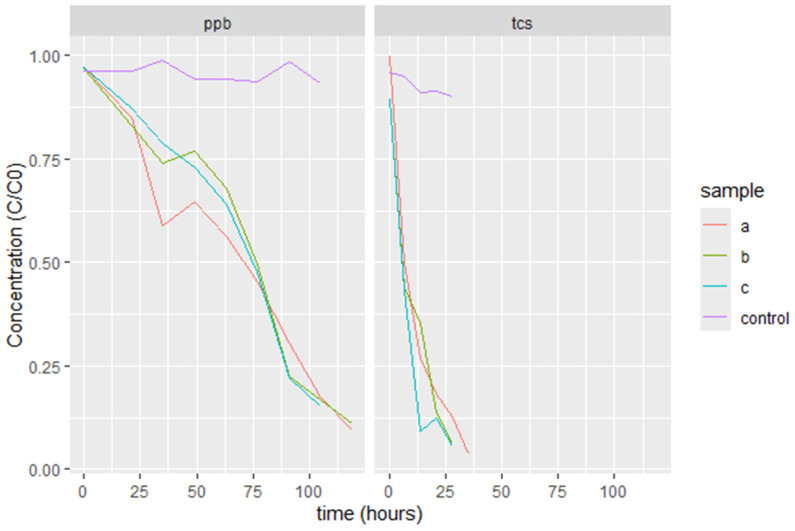
Biodegradation of triclosan and propylparaben at 2 mg/L over time (hours), with C_0_ as the initial concentration and C as the concentration at any given time.

So far, our research has focused on the exposure of TCS and PPB to bacteria and their distinct reactions in terms of developing antibiotic resistance. As shown in Figure 9, a rapid biodegradation of TCS was observed, and 28 h were required to achieve the complete disappearance of TCS. In the control, the removal is negligible. The triclosan concentration was reduced to 50% after 7 h, and the degradation rate decreased in the last 15 h of the experiment. In contrast, the biodegradation of PPB lasted more than 100 h until complete removal. The propylparaben concentration was reduced to 50% after 75 h, and surprisingly, the degradation rate increased in the last 25 h of the experiment. For both compounds, biodegradation can be an additional mechanism of resistance that can also result in resistance to other antibiotics.

PCR, Etest, and Biolog showed that isolates exposed for 10 cycles (D4 and D7 for example) to either TCS or PPB contained more ARG, and were resistant to more antibiotics. We hypothesized that isolates with antibiotic resistance acquired through co-selection can rapidly degrade TCS and PPB. However, the isolate exposed to PPB was shown to have a low degradation rate.

PCR, Etest, and Biolog showed that isolates exposed for 10 cycles (D4 and D7, for example) to either TCS or PPB contained more ARG, and were resistant to more antibiotics. We hypothesized that isolates with antibiotic resistance acquired through co-selection can rapidly degrade TCS and PPB. However, the isolate exposed to PPB was shown to have a low degradation rate.

While the resistance mechanisms of bacteria to micropollutants have been explored in detail over the years, the interplay between resistance and degradation remains largely overlooked [69]. The microbial degradation of TCS has not been reported frequently because it inhibits microbe growth [70]. As of 2022, a total of 19 bacterial strains capable of degrading TCS have been reported [71]. However, none of these strains have been evaluated for their potential to affect antibiotic-resistance genes. This study, therefore, emphasizes the distinction between bacterial resistance development and biodegradation capabilities. The decreased concentration of TCS and PPB in our studied microcosms is most likely a result of biological transformation rather than “removal” [72,73].

Propylparaben can be degraded into phenol and p-HBA and used as carbon sources by microorganisms [30,74], and specifically by the *Pseudomonas* strain [75]. Under aerobic conditions, the degradation pathway of parabens has two steps: firstly, the hydrolysis of the ester bond to produce p-HBA, followed by a decarboxylation to produce phenol [76]. Hydroxylation reactions of parabens form protocatechuate [77]. This compound can be further degraded in the β-ketoadipate pathway.

Studies have shown that TCS would rapidly be converted into transformation products [78]. It is degraded by the ammonia-oxidizing bacteria and heterotrophic bacteria in the water environment [71]. Biodegradation is mainly through oxygenation to open the aromatic ring, and through reductive dichlorination biotransformation. TCS transformation products, such as methyl-triclosan and carbanilides [79], however, are still able to induce endocrine disruption [80]. Transformation products like triclosan sulfate have the capacity to transform back into TCS [81]. Several potential pathways were described by [82], such as hydroxylation, where carbon–hydrogen is converted to a carbon–hydroxyl bond.

## 4. Conclusions

This study is the first to report on the complex relationship between bacterial resistance to antibiotics induced by antimicrobial chemicals and the biodegradation of antimicrobial substances by bacteria. For both triclosan and propylparaben, there is no significant difference in antibiotic resistance between the isolates obtained at different times of exposure. This suggests that the antibiotic-resistance gene is obtained rapidly, and once it is acquired, it is not proportional to the amount of micropollutant exposed. There is no noticeable correlation between the amount of ARGs and antibiotic resistance, as tested by Etest and PCR. The findings also indicate that TCS and PPB, even at environmentally relevant concentrations, can induce multidrug resistance. The identification of the isolates, together with the detection of genetic changes, provides a comprehensive analysis of the potential role of emerging chemicals in selecting for antibiotic resistance.

## Figures and Tables

**Table 1:** Bacterial mechanisms inducing co-selection, adapted from [40].

Mechanisms of Antibacterial Resistance	Type of Resistance	Co-Selection
Target site modifications	Acquired	No
Reduced permeability of the outer membrane	Natural	Yes
Increased efflux pumps	Natural	Yes
Enzymatic modification	Natural/acquired	No

**Table 2:** Antibiotics used and their mechanisms of action.

Antibiotic	Mechanisms of Action
TC = tetracycline	inhibits protein biosynthesis by targeting the ribosomal 30S subunits [44]
GM = gentamicin	inhibits protein synthesis and binds irreversibly to ribosomal 30S subunits [45]
TS = trimethoprim/sulfonamide	inhibits folic acid metabolism [44] by targeting the enzyme dihydropteroate synthase (DHPS) [46]
CL = ciprofloxacin	inhibits DNA replication by inhibiting bacterial DNA topoisomerase and DNA gyrase [47]
AM = ampicillin	inhibits bacterial cell wall synthesis by binding one or more of the binding proteins [48]
AC = amoxicillin	inhibits the biosynthesis and repair of the bacterial wall [49]

**Table 3:** Identification of the isolates obtained during the exposure experiment. It includes the results of the 16S rRNA bacterial sequencing, PCR analysis, and Etest. The section “Isolate details” contains the details of the exposure experiment (the type of chemical used and the concentration of exposure (conc)). The abbreviations of the Etest are as follows: TC = tetracycline, GM = gentamicin, T/S = trimethoprim/sulfamethoxazole, CI = ciprofloxacin, AM = ampicillin, AC = amoxicillin. Below each MIC value, it is written if the isolate is susceptible [S], intermediate [I], resistant [R], or [*] no inhibition zone.

Isolate Details				Identification		PCR (Presence or Absence)								Etest (MIC µg/mL)					
ID	Cycle	Chemical	Conc. (mg/L)		Most Similar Accession Number	fabV	tetG	qnrS	intI	blaCTX	ermB	sul1	Fab I	TC	GM	T/S	CI	AM	AC
D1	10	TCS	2	*Achromobacter insolitus*	CP026973.1	x		x	x			x	x	0.5 [S]	12 [I]	- [*]	0.5 [S]	1.5 [S]	1.5 [S]
D2	10	TCS	2	*Achromobacter insolitus*	CP026973.1	x		x	x			x	x	0.5 [S]	12 [I]	- [*]	0.75 [S]	1.5 [S]	1.5 [S]
D3	10	TCS	0.2	*Achromobacter insolitus*	CP026973.1			x			x	x	x	0.5 [S]	8 [I]	- [*]	0.5 [S]	1 [S]	1 [S]
D4	10	TCS	2	*Achromobacter insolitus*	CP026973.1	x	x	x	x			x		0.38 [S]	12 [I]	- [*]	1 [S]	1.5 [S]	1.5 [S]
D5	10	TCS	0.2	*Pseudomonas nitroreducens*	CP049140.1			x			x	x		4 [S]	0.75 [S]	- [*]	0.094 [S]	- [*]	- [*]
D6	10	TCS	2	*Pseudomonas nitroreducens*	CP049140.1	x	x	x	x		x	x		24 [R]	48 [R]	- [*]	1.5 [I]	1.5 [S]	1.5 [S]
DS8	5	TCS	2	*Pseudomonas* sp. *AU2510*	AY486376.1	x	x	x	x			x	x	3 [S]	16 [R]	- [*]	1.5 [I]	256 [S]	- [*]
DS9	5	TCS	2	*Pseudomonas nitroreducens*	CP049140.1		x	x	x			x	x	0.75 [S]	12 [I]	- [*]	0.75 [S]	6 [S]	12 [S]
D7	10	PPB	2	*Pseudomonas* sp. *BT5A*	LC097206.1	x	x	x			x	x	x	24 [R]	96 [R]	- [*]	6 [R]	1.0 [S]	1.5 [S]
D8	10	PPB	2	*Pseudomonas nitroreducens*	CP049140.1	x	x	x	x	x		x		24 [R]	12 [I]	- [*]	1.0 [S]	1.5 [S]	1.5 [S]
D9	10	PPB	2	*Pseudomonas nitroreducens*	CP049140.1	x		x	x	x		x	x	4 [S]	0.75 [S]	- [*]	0.094 [S]	- [*]	- [*]
D10	10	PPB	2	*Pseudomonas nitroreducens*	CP049140.1	x		x	x			x	x	3 [S]	0.75 [S]	- [*]	0.064 [S]	- [*]	96 [R]
D11	10	PPB	0.2	*Pseudomonas nitroreducens*	CP049140.1	x	x	x	x	x	x	x	x	4 [S]	0.5 [S]	- [*]	0.064 [S]	3 [S]	24 [I]
D12	5	PPB	2	*Pseudomonas nitroreducens*	CP049140.1		x	x			x	x		3 [S]	0.5 [S]	4 [R]	0.094 [S]	256 [R]	64 [R]
D13	5	PPB	2	*Pseudomonas* sp. *BT5A*	LC097206.1		x	x				x		6 [I]	1 [S]	6 [R]	0.064 [S]	256 [R]	64 [R]
D14	5	PPB	2	*Pseudomonas nitroreducens*	CP049140.1	x	x	x			x	x		1.0 [S]	8 [I]	12 [R]	0.75 [S]	- [*]	8 [S]

**Table 4:** Half-maximal inhibitory concentration (IC_50_) of the selected isolates for triclosan and propylparaben and their related MIC value.

	TCS			PPB		
Strain	IC_50_ (mg/L)	R^2^	MIC (mg/L)	IC_50_ (mg/L)	R^2^	MIC (mg/L)
D4	16.61	0.918	64.0	191.0	0.919	512
D7	18.63	0.894	128.0	75.0	0.974	512
D13	182.1	0.0891	>256	193.4	0.918	800

## Data Availability

All data have been made available.

## Supplementary Materials

The following supporting information can be downloaded at: https://www.mdpi.com/article/10.3390/jox15020056/s1. Table S1: Chemical characteristics of micropollutants. Table S2: List for a universal procedure to itemize chemicals for synthetic greywater. Table S3: The results of the quality control Etest. Table S4: Used primers, their sequences, and mode of action. Figure S1: Growth curve of D4 grown on propylparaben. Figure S2: Growth curve of D7 grown on propylparaben. Figure S3: Growth curve of D13 grown on propylparaben. Figure S4: Growth curve of D4 grown on triclosan. Figure S5: Growth curve of D7 grown on triclosan. Figure S6: Growth curve of D13 grown on triclosan. References [26,83,84,85,86,87,88,89,90,91,92,93,94,95,96] are cited in the Supplementary Materials.

## Abbreviations

The following abbreviations are used in this manuscript:

EMP	Emerging micropollutant
TCS	Triclosan
PPB	Propylparaben
MIC	Minimum inhibitory concentration
HGT	Horizontal gene transfer
OD	Optical density
PCR	Polymerase chain reaction
