# Supplementary material for: Resistance and Biodegradation of Triclosan and Propylparaben by Isolated Bacteria from Greywater
Source: J Xenobiot. 2025 Apr 15;15(2):56. doi: 10.3390/jox15020056 (PMC12028367; doi:10.3390/jox15020056)
Supplement: Supplementary file 1 [file jox-15-00056-s001.zip › jox-3540195-supplementary.pdf]

Resistance and biodegradation of triclosan and propylparaben by isolated bacteria from greywater

Daniella Itzhari[1], Joseph Nzeh[1] and Zeev Ronen[1] *

[1]Zuckerberg Institute for Water Research, The Jacob Blaustein Institutes for Desert Research, Ben Gurion University of the Negev, Beersheba 8499000, Israel; vanderro@post.bgu.ac.il
* Correspondence: zeevrone@bgu.ac.il

Supplementary Material

Table S1: Chemical characteristics of micropollutants

**Compound**	Molecular formula	Chemical structure	Molecular weight (g/mol)
Propylparaben	$C_{10}H_{12}O_3$	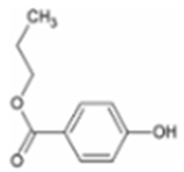	180.2
Triclosan	$C_{12}H_7Cl_3O_2$	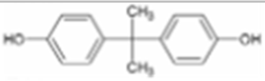	289.5

Table S2: list for an universal procedure to itemize chemicals for synthetic greywater.

Chemical name	Chemical formula	Molar mass (g/mol)	Concentration (mg/l)	Composition percentages
**Bentonite**	$Al_2O_3{\cdot}4SiO_2{\cdot}H_2O$	180.1	100	
**Cellulose**	$(C_6H_{10}O_5)n$	162.14	100	C (44.45%), H (6.22%) and O (49.34%)
**Humic acid**	$C_{187}H_{186}O_{89}N_9S_1$	4015.55	20	C (55.90%), H (4.67%), O (35.46%), N (4.67%) and S (0.80%)
**Sodium chloride**	NaCl	58.44	120	Cl (60.66%) and Na (39.34%)
**Sodium hydrogen carbonate**	$NaHCO_3$	84.01	85	C (14.30%), H (1.20%), Na (27.37%) and O (57.14%)
**Calcium chloride**	$CaCl_2$	147.02	55	Ca (36.11%) and Cl (63.89%)
**Potassium nitrate**	KNO_3	101.1	90	K (38.67%), N (13.85%) and O (47.48%)
**Calcium nitrate**	$Ca(NO_3)_2$	164.09	150	Ca (24.43%), N (17.07%) and O (58.50%)
**Magnesium sulphate**	$MgSO_4$	120.37	240	Mg (20.19%), S (26.64%) and O (53.17%)
**Monopotassium phosphate**	KH_2PO_4	136.09	85	H (1.48%), K (28.73%), O (47.03%) and P (22.76%)
**Iron(iii)chloride**	$FeCl_3$	162.2	50	Fe (34.43%) and Cl (65.57%)
**Boric acid**	H_3BO_3	61.83	3	H (4.89%), B (17.48%) and O (77.63%)
**Manganese(ii)chloride**	$MnCl_2$	125.84	3,2	Cl (56.34%) and Mn (43.66%)
**Zinc sulphate**	$ZnSO_4$	161.44	15	O (39.64%), S (19.86%) and Zn (40.50%)
**Ammonium molybdate tetrahydrate**	$(NH_4)_6Mo_7O_{24}$	1163.94	0.35	H (2.08%), Mo (57.71%), N (7.22%) and O (32.99%)
**Sodium phosphate monobasic**	H_2NaPO_4	119.98	250	H (1.68%), Na (19.16%), O (53.34%) and P (25.82%)

Table S3. The results of the quality control Etest.

control		TC	GM	T/S	CI	AM	AC
1	E. coli 25922	0.4	0.5	0	0	2	4
2	P. putida	1	0.1	*	0.02	32	16

Table S4. Used primers, their sequences and mode of action

**Primer**	**Primer sequence**	**Mechanisms of antibiotic resistance**
tetG	F: GCACGCTGGTTTGGCTACA<br>R: TGGCTGTGATTAGTCTCCTTGA	modifying the ribosomal 30S subunits [43]
qnrS	F: GACGTGCTAACTTGCGTG<br>R: TGGCATTGTTGGAAACTT	Alterations in target enzymes [83]
sul1	F: CGCACCGGAAACATCGCTGCAC<br>R: TGAAGTTCCGCCGCAAGGCTCG	acquisition of alternative of the DHPS enzymes [45]
FabI	F: AGACGGATCGGCGATAACAA<br>R: GCTACTCACAGCCAGGTTGA	catalyse the reduction of *trans*-2-acyl-ACPs (an enoyl-ACP) to the fully saturated acyl-ACP species [84]
FabV	F: CATACAGCGGCGTGTGTTAC<br>R: CCTTGCCAATCCCTAGTCCC	Same as FabI
intI	F: GATCGGTCGAATGCGTGT<br>R: GCCTTGATGTTACCCGAGAG	Class 1 integrons acts as a pool of antimicrobial resistance gene cassettes [85]
ermB	F: CGTGCGTCTGACATCTATCTGA<br>R: CTGTGGTATGGCGGGTAAGTT	modification of the ribosomal target by methylation and active efflux of the drug [86]
blaCTX	F: CGTCACGCTGTTGTTAGGAA<br>R: CGCTCATCAGCACGATAAAG	enzymes break down beta-lactam antibiotic proteins [87]

The Minimum Inhibitory Concentration (MIC) is defined as the lowest concentration of antimicrobial agent at which no growth is detected [88], [89]. MIC of PPB for Gram-positive bacteria has been reported to range between 250-2000 ug/mL (with most strains MIC of 500ug/mL), and for Gram-negative bacteria between 500-2000 ug/mL (with most strains MIC of 2000 ug/mL) [90] More examples are strains reported to have a MIC to PPB between 1250-2500 ug/mL [91], and at the concentration of 881 μg/mL [92] MIC of TCS is much lower at the range of between 0.025 and 1 mg/L [93], 2–4 mg/L [94], 64 μg/mL [95], 64.0 to 1,024.0 ug/ml [96], 10 mg/L [26].

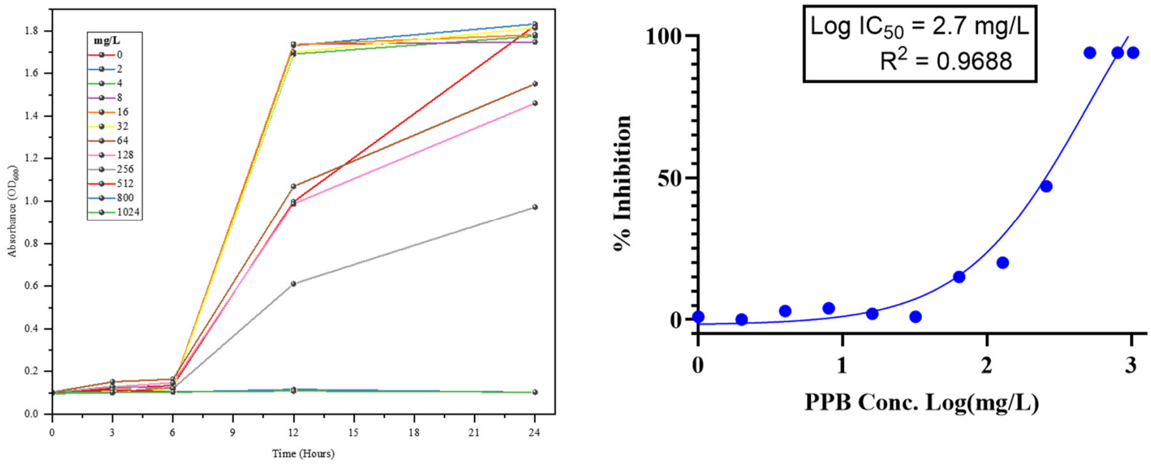

Figure 1: Growth curve of **D4** grown on Propylparaben

1

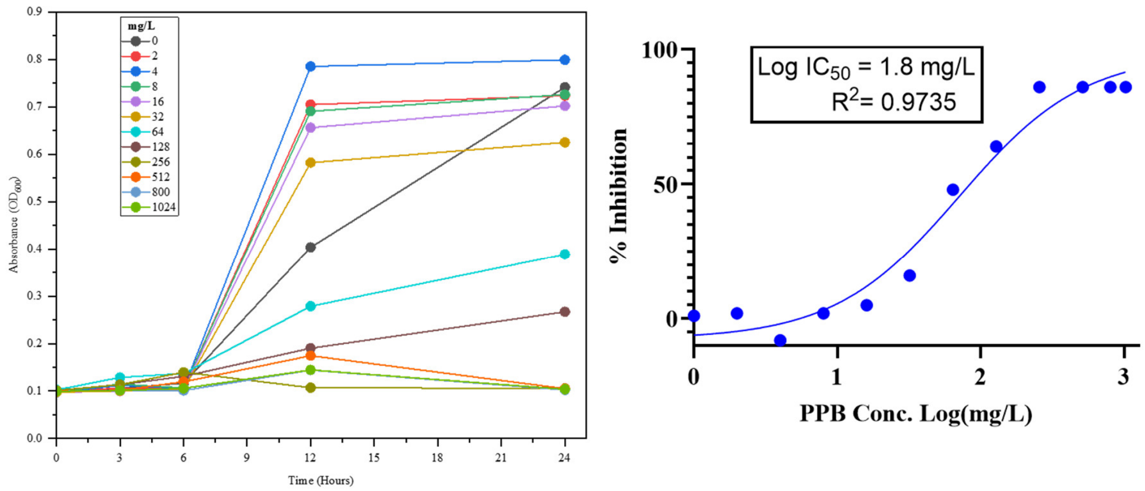

Figure 2: Growth curve of **D7** grown on Propylparaben

2

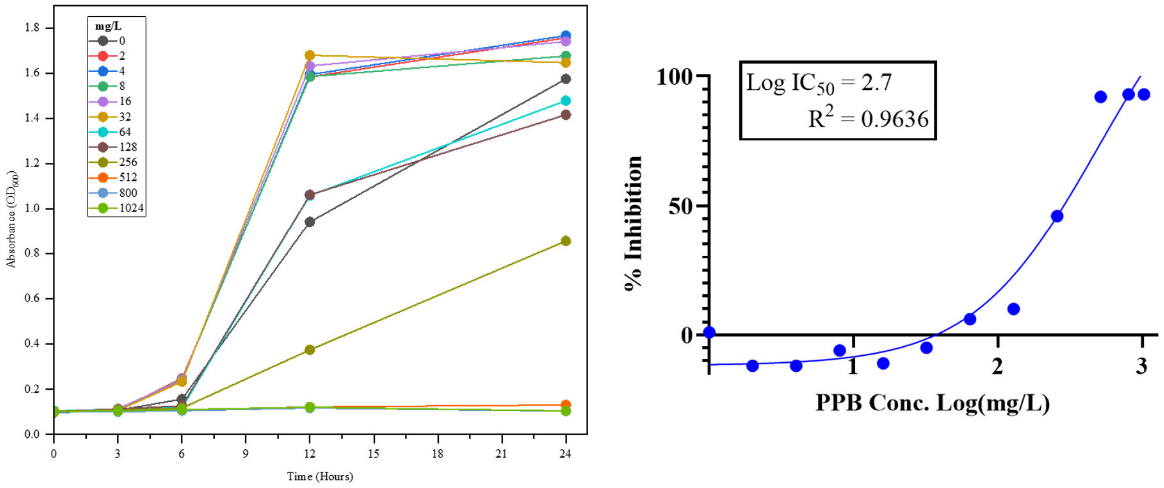

Figure 3: Growth curve of **D13** grown on Propylparaben 3

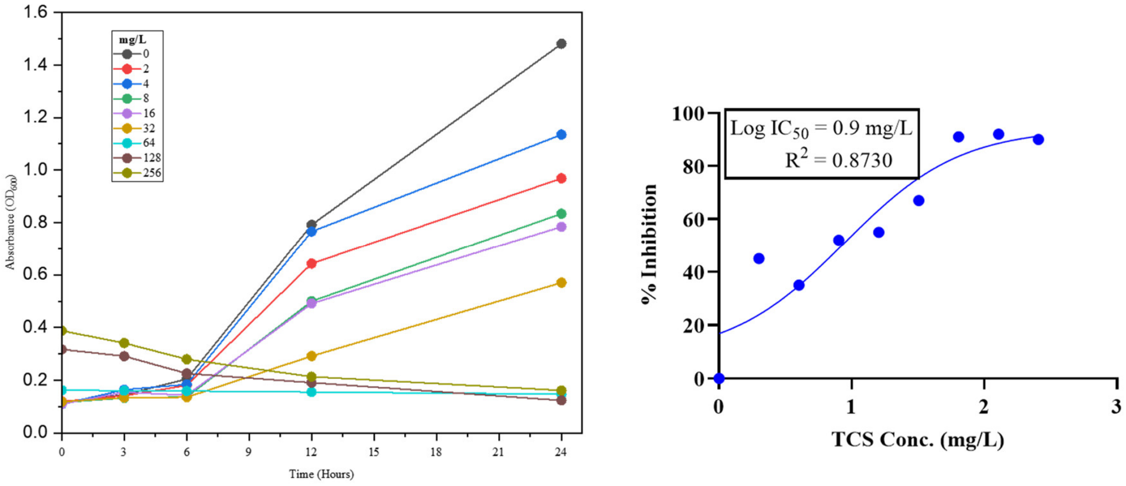

Figure 4: Growth curve of **D4** grown on Triclosan 1

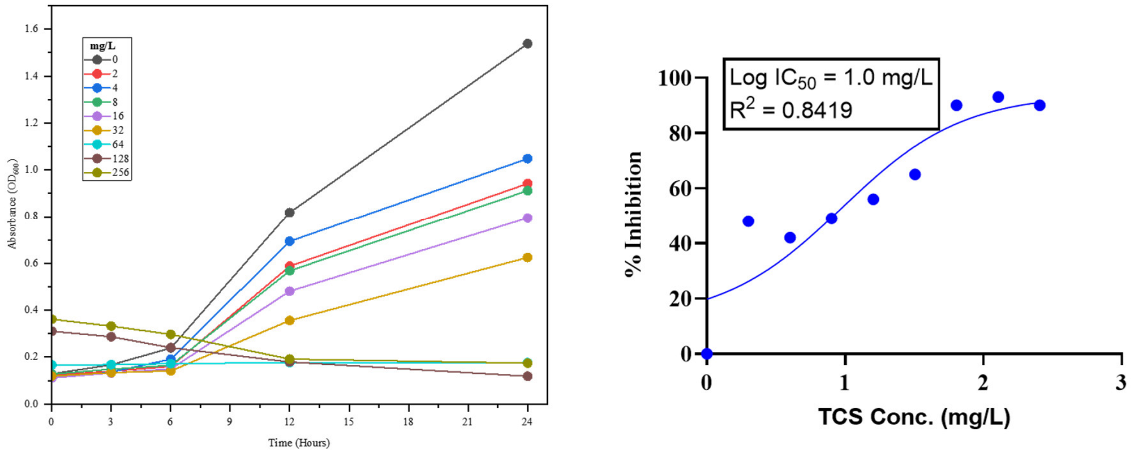

Figure 5: Growth curve of **D7** grown on Triclosan 2

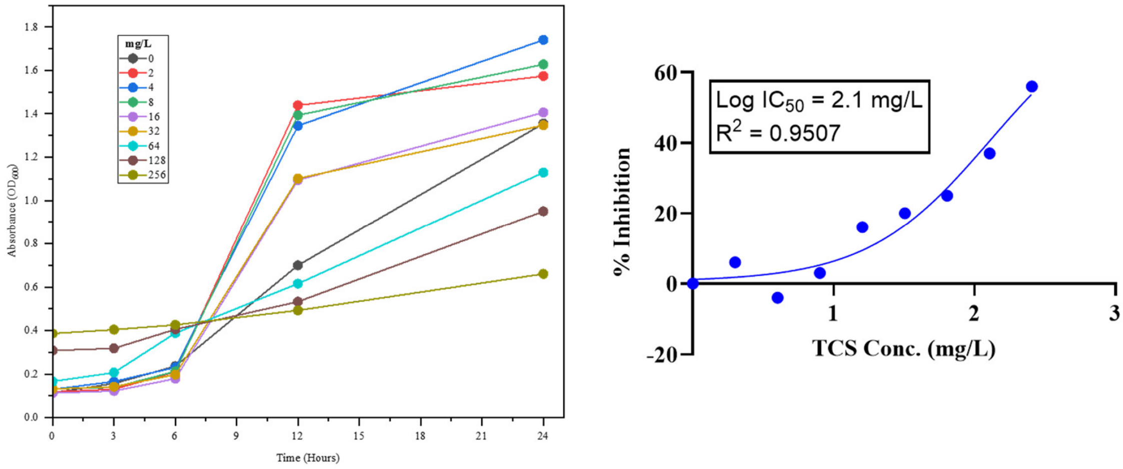

Figure 6: Growth curve of **D13** grown on Triclosan 3
